# Somatic disease burden and depression risk in late life: a community-based study

**DOI:** 10.1017/S2045796024000064

**Published:** 2024-02-08

**Authors:** Federico Triolo, Davide Liborio Vetrano, Linnea Sjöberg, Amaia Calderón-Larrañaga, Martino Belvederi Murri, Laura Fratiglioni, Serhiy Dekhtyar

**Affiliations:** 1 Aging Research Center, Department of Neurobiology, Care Sciences and Society, Karolinska Institutet and Stockholm University, Stockholm, Sweden; 2 Stockholm Gerontology Research Center, Stockholm, Sweden; 3 Institute of Psychiatry, Department of Neuroscience and Rehabilitation, University of Ferrara, Ferrara, Italy

**Keywords:** disease patterns population-based, late life depression, multimorbidity, psychosomatic medicine

## Abstract

**Aims:**

Co-occurring somatic diseases exhibit complex clinical profiles, which can differentially impact the development of late-life depression. Within a community-based cohort, we aimed to explore the association between somatic disease burden, both in terms of the number of diseases and their patterns, and the incidence of depression in older people.

**Methods:**

We analysed longitudinal data of depression- and dementia-free individuals aged 60+ years from the population-based Swedish National Study on Aging and Care in Kungsholmen. Depression diagnoses were clinically ascertained following the Diagnostic and Statistical Manual of Mental Disorders, Fourth Edition Text Revision over a 15-year follow-up. Somatic disease burden was assessed at baseline through a comprehensive list of chronic diseases obtained by combining information from clinical examinations, medication reviews and national registers and operationalized as (i) disease count and (ii) patterns of co-occurring diseases from latent class analysis. The association of somatic disease burden with depression incidence was investigated using Cox models, accounting for sociodemographic, lifestyle and clinical factors.

**Results:**

The analytical sample comprised 2904 people (mean age, 73.2 [standard deviation (SD), 10.5]; female, 63.1%). Over the follow-up (mean length, 9.6 years [SD, 4 years]), 225 depression cases were detected. Each additional disease was associated with the occurrence of any depression in a dose–response manner (hazard ratio [HR], 1.16; 95% confidence interval [CI]: 1.08, 1.24). As for disease patterns, individuals presenting with sensory/anaemia (HR, 1.91; 95% CI: 1.03, 3.53), thyroid/musculoskeletal (HR, 1.90; 95% CI: 1.06, 3.39) and cardiometabolic (HR, 2.77; 95% CI: 1.40, 5.46) patterns exhibited with higher depression hazards, compared to those without 2+ diseases (multimorbidity). In the subsample of multimorbid individuals (85%), only the cardiometabolic pattern remained associated with a higher depression hazard compared to the unspecific pattern (HR, 1.71; 95% CI: 1.02, 2.84).

**Conclusions:**

Both number and patterns of co-occurring somatic diseases are associated with an increased risk of late-life depression. Mental health should be closely monitored among older adults with high somatic burden, especially if affected by cardiometabolic multimorbidity.

## Introduction

Late-life depression, a common and burdensome condition at both individual and societal levels, is strongly impacted by the somatic disease burden (Alexopoulos, 2019). Individuals with single chronic diseases are not only at increased risk of developing depression in late life but also of experiencing a worse clinical course (Hegeman *et al.*, 2017; Huang *et al.*, 2010). Such effects may be attributed to the complex pathophysiology of depression that to a certain extent involves the same biological, psychosocial and care-related factors as several chronic diseases (Gold *et al.*, 2020). As a result, somatic comorbidity in older individuals with depression is associated with functional decline, higher healthcare utilization and shorter survival (Quinones *et al.*, 2018). Therefore, understanding how somatic health shapes depression risk represents a critical step towards improved prevention and care for mental health in an aging society.

The coexistence of multiple chronic diseases, i.e., multimorbidity, is a key contributor to the high health heterogeneity and clinical complexity observed in older adults (Marengoni *et al.*, 2011; Santoni *et al.*, 2015; Vetrano *et al.*, 2018a). The clinical challenges posed by multimorbidity are both considerable and poorly addressed in clinical guidelines, as multimorbid persons are routinely excluded from randomized controlled trials (Skou *et al.*, 2022; Vetrano *et al.*, 2018a; Whitty *et al.*, 2020). While multimorbidity has been mostly operationalized through quantitative measures (i.e., count of diseases and presence of ≥2 diseases), evidence suggests that diseases do not co-occur randomly, but in patterns, based on shared mechanisms and risk factors (Prados-Torres *et al.*, 2014; Vetrano *et al.*, 2020). Characterizing individuals based on their patterns of co-occurring diseases may better capture different health trajectories in old age (Skou *et al.*, 2022; Vetrano *et al.*, 2020). For instance, cardiovascular and neuropsychiatric patterns of multimorbidity have been consistently previously described and linked with several adverse outcomes, including frailty, unplanned hospitalizations, dementia and higher mortality (Akugizibwe *et al.*, 2020; Grande *et al.*, 2021; Tazzeo *et al.*, 2021; Vetrano *et al.*, 2022).

While an association between somatic disease burden and the development of depression in late life has been previously suggested (Triolo *et al.*, 2020), the role played by both the number and the combinations of co-occurring diseases has been rarely investigated in the same population. Previous studies have mostly used symptom rating scales rather than clinical diagnoses to ascertain depression, analysed cohorts of middle-aged individuals or employed a limited number of chronic diseases to derive multimorbidity patterns (Ronaldson *et al.*, 2021; Triolo *et al.*, 2020; Yao *et al.*, 2020). Notably, few have considered the implication of disease patterns for depression among individuals already affected by multimorbidity, a highly prevalent condition in old age (Calderón-Larrañaga *et al.*, 2017). Doing so, will help better inform clinical recommendations and improve care provision for those with complex health profiles (Skou *et al.*, 2022).

This study aims to investigate the association between somatic disease burden and the risk of depression within a community-based cohort of older individuals. We hypothesize that somatic disease burden is associated with late-life depression not only in terms of the number of diseases but also in relation to different patterns of co-occurring diseases.

## Materials and methods

### Study population

This study was carried out within the Swedish National Study on Aging and Care in Kungsholmen (SNAC-K, http://www.snac-k.se/), an ongoing population-based study of individuals aged 60 years or older living in the Kungsholmen area of Stockholm, Sweden (Lagergren *et al.*, 2004). SNAC-K used stratified random sampling and included cohorts aged 60, 66, 72, 78 and 81+ years at baseline. Of the eligible individuals, 3363 (73% participation rate) accepted to participate in the baseline examination (2001–2004), which comprised an extensive health assessment carried out by healthcare professionals. After excluding individuals with prevalent major or minor depression (*n* = 222), dementia (*n* = 201) or with missing information on depression at baseline (*n* = 36), the study population comprised 2904 participants who were regularly followed-up every 6 years for the younger age cohorts (60–72 years) or every 3 years for the older age cohorts (78+ years). In this study, we used longitudinal data up to wave 5 (2016–2019), resulting in a follow-up of up to 15 years (Figure S1).

### Chronic somatic diseases at baseline

The assessment of chronic diseases in SNAC-K was carried out through a previously described algorithm that integrates detailed health information from multiple sources, including SNAC-K medical examination, blood tests, medication reviews and inpatient and outpatient records via linkage to the Swedish National Patient Register (Calderón-Larranaga *et al.*, 2017). A clinically driven selection of 918 codes of chronic diseases from the International Classification of Diseases, 10th revision (ICD-10) was compiled by a multidisciplinary team of clinicians and researchers and grouped into a comprehensive list of 60 disease categories with relevance for old age (Calderón-Larrañaga *et al.*, 2017). After discarding psychiatric conditions and dementia, 54 somatic diseases (Table S1) were used to construct, for each individual, two measures of somatic disease burden at baseline: (i) counts of chronic somatic diseases in the complete sample and (ii) patterns of chronic somatic diseases in a subsample with multimorbidity obtained through latent class analysis (LCA) (see statistical analyses section for more information).

### Depression assessment at follow-up

Depression was defined by the development of either major or minor depression over the 15-year follow-up. The diagnoses were ascertained in accordance with the Diagnostic and Statistical Manual of Mental Disorders, Fourth Edition Text Revision (DSM-IV-TR) (American Psychiatric Association, 2000). We employed a previously described procedure to derive information on the nine DSM-IV-TR diagnostic criteria using selected depressive items of the Comprehensive Psychopathological Rating Scale (CPRS) (Sjöberg *et al.*, 2017). The CPRS is an extensive scale of psychiatric symptoms and signs carried out during SNAC-K medical assessments (Åsberg *et al.*, 1978). Major and minor depression were defined by the presence of at least five symptoms or two-to-four symptoms, respectively, with the requirement of one being a core symptom (low mood or loss of interest) (American Psychiatric Association, 2000).

### Covariates

In the analyses, we accounted for sociodemographic factors (age, sex, attained formal education [high school and below, university] and civil status [unmarried/single, married]), health behaviours (alcohol consumption [no or occasional, light to moderate, heavy drinking], smoking [ever, never]), disease severity (proxied by the presence of malnutrition [i.e., body mass index at baseline <18.5 kg/m^2^]) and history of depression (presence, absence). In sensitivity analyses, we further considered participants’ global cognitive status assessed using Mini Mental State Examination (MMSE), use of antidepressants (presence, absence) and baseline depressive symptoms based on the Montgomery–Åsberg Depression Rating Scale (MADRS) (Montgomery and Åsberg, 1979). All covariates were assessed at baseline.

### Statistical analyses

#### Multimorbidity patterns from LCA

Baseline multimorbidity patterns were estimated using LCA, which allows the identification of mutually exclusive groups of individuals sharing similar patterns of co-occurring somatic diseases. LCA was performed on individuals with at least two diseases, the most commonly used definition for the presence of multimorbidity (Skou *et al.*, 2022). This approach ensured the consistency of a minimum disease load (2+ diseases) in the subsequent analysis of disease patterns. In line with previous research, diseases with a prevalence below 2% in the study population were excluded to reduce statistical noise, with 34 somatic diseases being featured in the model (Table S1) (Vetrano *et al.*, 2020). The optimal number of classes was determined based on the model fit using the adjusted Bayesian Information Criterion (Figure S2), while ensuring that the suggested classes were not trivial in size (i.e., identifying ≤2% of the study population). Following previous work, we considered diseases as defining features of each class if they fulfilled two conditions: an observed/expected ratio ≥2 (O/E) and exclusivity >25% (Table S2) (Vetrano *et al.*, 2020). O/E was calculated as the prevalence of a specific disease in one class over the prevalence of the disease in the study population. Exclusivity was calculated as the proportion of individuals with the disease in the class over the total number of individuals with the disease in the study population (Vetrano *et al.*, 2020). After estimating class membership probabilities, individuals were assigned to the class with the highest probability.

#### Associations with incident depression

The associations between somatic disease count and patterns with incident depression were estimated using Cox proportional hazard regression models. Follow-up time was defined as the time between study entry and depression diagnosis, loss to follow-up or study end, whichever occurred first. The proportional hazards assumption was tested with Schoenfeld residuals, and covariates violating it were modelled using time-varying effects through interaction with the timescale. Number of somatic diseases was modelled as a continuous variable, and both its linear and non-linear associations with depression incidence were examined. The non-linear association was visualized graphically using three restricted cubic splines at the 10th, 50th and 90th percentiles of the somatic disease count variable. The association between disease patterns and depression was estimated in the (i) full study population using individuals with no multimorbidity as the reference group and (ii) multimorbid subpopulation using individuals in the unspecific pattern as the reference group, as done in previous studies using a similar methodology (Akugizibwe *et al.*, 2020; Grande *et al.*, 2021; Tazzeo *et al.*, 2021). For all models, we provide hazard ratios (HRs) with 95% confidence intervals (95% CIs) with basic (age, sex and education) and full (age, sex, education, civil status, alcohol consumption, smoking, malnutrition and history of depression) adjustment.

#### Sensitivity analyses

To ensure that the associations between somatic diseases and depression were not driven by cognitive decline or incipient dementia, we repeated all analyses excluding participants with MMSE <24 at baseline or incident cases of dementia in the first 6 years of follow-up. To minimize the potential effect of depressive symptomatology at baseline, analyses were additionally adjusted for MADRS score. Last, we repeated the analyses adjusting for antidepressant use to mitigate the potential misclassification of baseline depressive status due to treatment.

A two-tailed *p*-value < 0.05 was considered statistically significant. All analyses were carried out with STATA 17 and R (version 4.2.1).

## Results

### Baseline descriptives and disease pattern characterization

The analytical sample comprised 2904 dementia and depression-free participants, with a mean age of 73.2 years and 63% being women. It included individuals without multimorbidity (15%), as well as those with two or more somatic diseases distributed across five disease patterns: *unspecific* (25%), *thyroid/musculoskeletal* (19%), *sensory/anaemia* (18%), *metabolic* (13%) and *cardiometabolic* (10%).

Details of LCA fit and of the diseases characterizing each pattern are reported in Figure S2 and Table 1, respectively (see Table S2 for a complete list of characterizing diseases). Baseline descriptives according to disease patterns are reported in Table 2.

**Table 1.** Disease pattern characterization

Somatic disease patterns	Characterizing diseases*	Prevalence within the pattern (%)
*Unspecific*	–	–
*Metabolic*	Obesity	45.3
	Diabetes	35.2
*Sensory/anaemia*	Deafness, hearing impairment	30.7
	Anaemia	29.0
*Thyroid/MSK*	Osteoarthritis and other degenerative joint diseases	36.9
	Thyroid diseases	26.4
	Osteoporosis	20.2
*Cardiometabolic*	Heart failure	75.6
	Ischemic heart disease	56.4
	Atrial fibrillation	43.3
	Anaemia	30.6
	Diabetes	21.8
	Cerebrovascular disease	20.2

* Diseases with observed/expected ratio ≥2 and exclusivity >25%. Only characterizing disease with prevalence over 20% within each pattern is reported, see Table S2 for the complete disease list.

**Table 2.** Baseline descriptive characteristics of the study population by disease patterns

	Total	No MM	Unspecific	Metabolic	Sensory/anaemia	Thyroid/MSK	Cardiometabolic
	*N* = 2904	*N* = 429, 15%	*N* = 716, 25%	*N* = 369, 13%	*N* = 514, 18%	*N* = 569, 19%	*N* = 307, 10%
Age, mean (SD)	73.2 (10.5)	65.0 (6.7)	69.3 (8.3)	71.4 (8.9)	80.6 (9.5)	73.4 (9.6)	83.2 (8.7)
Sex (women), *n* (%)	1832 (63.1%)	230 (53.6%)	450 (62.8%)	172 (46.6%)	326 (63.4%)	455 (80.0%)	199 (64.8%)
Education (university), *n* (%)	1017 (35.1%)	225 (52.6%)	276 (38.5%)	127 (34.4%)	152 (29.7%)	184 (32.5%)	53 (17.4%)
Marital status (not married), *n* (%)	1559 (53.9%)	168 (39.3%)	334 (46.6%)	180 (48.9%)	323 (63.1%)	348 (61.5%)	206 (67.5%)
Alcohol consumption							
(no or occasional use), *n* (%)	966 (33.5%)	70 (16.4%)	166 (23.2%)	116 (31.8%)	231 (45.5%)	218 (38.7%)	165 (54.6%)
(light to moderate use), *n* (%)	1446 (50.2%)	285 (66.6%)	400 (55.9%)	189 (51.8%)	218 (42.9%)	241 (42.8%)	113 (37.4%)
(heavy use), *n* (%)	470 (16.3%)	73 (17.1%)	150 (20.9%)	60 (16.4%)	59 (11.6%)	104 (18.5%)	24 (7.9%)
Smoking (ever), *n* (%)	409 (14.2%)	76 (17.9%)	125 (17.6%)	48 (13.1%)	49 (9.6%)	83 (14.7%)	28 (9.2%)
Somatic diseases, mean (SD)	3.7 (2.3)	0.8 (0.4)	3.0 (1.0)	3.7 (1.6)	4.6 (1.8)	4.0 (1.7)	7.3 (2.3)
MADRS, mean (SD)	2.1 (2.8)	1.6 (2.5)	1.7 (2.6)	1.9 (2.7)	2.5 (2.8)	2.4 (3.0)	3.0 (3.3)
Malnutrition, *n* (%)	64 (2.3%)	1 (0.2%)	7 (1.0%)	6 (1.7%)	23 (4.8%)	18 (3.3%)	9 (3.3%)
History of depression, *n* (%)	343 (11.9%)	53 (12.5%)	87 (12.3%)	38 (10.4%)	54 (10.6%)	86 (15.2%)	25 (8.3%)
Antidepressant use, *n* (%)	208 (7.2%)	12 (2.8%)	40 (5.6%)	28 (7.6%)	43 (8.4%)	60 (10.5%)	25 (8.2%)
MMSE, mean (SD)	28.6 (1.9)	29.2 (1.7)	29.0 (1.5)	28.9 (1.4)	27.9 (2.3)	28.8 (1.7)	27.8 (2.3)

All tests across patterns were statistically significant. SD: standard deviation; MM: multimorbidity; MSK: musculoskeletal. MADRS: Montgomery–Åsberg Depression Rating Scale; MMSE: Mini Mental State Examination. Missing: education (*n* = 9), marital status (*n* = 9), alcohol consumption (*n* = 22), smoking (*n* = 26), malnutrition (*n* = 115), history of depression (*n* = 27), MADRS (*n* = 73), MMSE (*n* = 3) and antidepressant use (*n* = 4).

Compared to the entire study population, those without multimorbidity (*n* = 429, 15%) were significantly more likely to be younger, male, more educated, married and with higher global cognitive function. In the multimorbid subpopulation, those characterized by the *unspecific* pattern tended to be younger, more educated and married in comparison to individuals with other disease patterns, especially the *cardiometabolic*, which comprised the oldest participants with the least advantageous background characteristics.

### Number of somatic diseases and depression risk

Of the 2904 eligible baseline participants, 749 (26%) were lost to attrition due to death (*n* = 368) or dropout (*n* = 338) before the first follow-up or missing information on depression status in any follow-up (*n* = 43) (Figure S1). Participants not included in the longitudinal analyses tended to be older, less educated, unmarried, with worse somatic health and cognition, and were more likely to display a *cardiometabolic* or *sensory/anaemia* pattern (data not shown).

During the 15-year follow-up, 225 depression cases were detected among 2155 participants, and the average follow-up time was 9.6 years (standard deviation, 4 years). In a multi-adjusted model, each additional somatic disease was associated with a 16% increase in the hazard of depression (HR, 1.16; 95% CI: 1.08–1.24).

**Figure 1.**
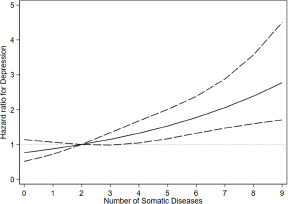
Association of the number of somatic diseases with risk of depression. The number of somatic diseases was modelled with three cubic restricted splines at 10th, 50th and 90th percentile. Hazard ratios obtained from Cox proportional hazards model adjusted for age, sex, education, marital status, alcohol consumption, smoking, malnutrition and history of depression. The solid curved line depicts point estimates, dashed lines indicate the corresponding 95% confidence intervals, while the dotted line presents the reference line.

We further explored the non-linear association between the number of somatic diseases and depression risk, which is visually presented in Fig. 1. Compared to individuals with two somatic diseases, the presence of at least four somatic diseases was associated with a statistically significant increased hazard of depression (HR for four diseases, 1.31 and 95% CI: 1.06–1.63; HR for five diseases, 1.49 and 95% CI: 1.16–1.92).

### Somatic disease patterns and depression risk

Table 3 reports the association between somatic disease patterns and incident depression.

**Table 3.** Association between disease patterns and risk of depression

Total sample	Incidence rate (95% CI)	Model 1 Events: 222 At risk: 2152	Model 2 Events: 210 At risk: 2075
No MM	4.1 (2.5−6.6)	Reference	Reference
Unspecific	9.2 (7.1−11.9)	1.74 (0.99−3.05)	1.65 (0.94−2.90)
Metabolic	6.6 (4.1−10.7)	1.15 (0.58−2.30)	1.06 (0.53−2.15)
Sensory/anaemia	17.5 (13.1−23.3)	**2.19 (1.19−4.03)**	**1.91 (1.03−3.53)**
Thyroid/MSK	13.4 (10.4−17.4)	**2.09 (1.17−3.73)**	**1.90 (1.06−3.39)**
Cardiometabolic	26.3 (18.3−37.9)	**3.16 (1.64−6.12)**	**2.77 (1.40−5.46)**

Multimorbid subpopulation	Incidence rate (95% CI)	Model 1 Events: 206 At risk: 1798	Model 2 Events: 194 At risk: 1727
Unspecific	9.2 (7.1−11.9)	Reference	Reference
Metabolic	6.6 (4.1−10.7)	0.67 (0.39−1.15)	0.66 (0.38−1.15)
Sensory/anaemia	17.5 (13.1−23.3)	1.27 (0.83−1.93)	1.17 (0.76−1.79)
Thyroid/MSK	13.4 (10.4−17.4)	1.20 (0.82−1.75)	1.15 (0.78−1.70)
Cardiometabolic	26.3 (18.3−37.9)	**1.84 (1.13−2.98)**	**1.71 (1.02−2.84)**

IR (95% CI): incident rates per 1000 person-years with 95% confidence intervals; MSK: musculoskeletal; Model 1: age, sex and education; Model 2: age, sex, education, marital status, alcohol consumption, smoking, malnutrition and history of depression. Missing: education (*n* = 3), marital status (*n* = 2), alcohol consumption (*n* = 14), smoking (*n* = 10), malnutrition (*n* = 44) and history of depression (*n* = 19). Bold indicates *p* < 0.05.

In the multi-adjusted models, individuals with *sensory/anaemia* (HR, 1.91; 95% CI: 1.03–3.53), *thyroid/musculoskeletal* (HR, 1.90; 95% CI: 1.06–3.39) or *cardiometabolic* (HR, 2.77; 95% CI: 1.40–5.46) disease patterns exhibited an increased hazard of depression, as compared to people without multimorbidity. In the subpopulation of individuals with multimorbidity, relative to the *unspecific* disease pattern, the *cardiometabolic* pattern remained the only one independently associated with a higher hazard of depression (HR, 1.71; 95% CI: 1.02–2.84).

### Sensitivity analyses

All results were preserved after excluding participants with baseline MMSE <24 or with dementia onset during the first 6 years of follow-up (Table S3). The additional adjustment for baseline MADRS score attenuated the strength of some of the associations, without changing the overall pattern of results (Table S4). Last, adjustment for antidepressant use did not alter the results (Table S5).

## Discussion

In this large sample of community-dwelling older individuals followed for 15 years, we observed that a greater number of somatic diseases was associated with a higher incidence of depression. Furthermore, *sensory/anaemia, thyroid/musculoskeletal* and *cardiometabolic* patterns of co-occurring somatic diseases were associated with a higher hazard of depression, compared to individuals with no multimorbidity. Among the multimorbid individuals, those presenting with the *cardiometabolic* pattern were characterized by an increased depression incidence compared to those in the *unspecific* one.

### Number of somatic diseases and depression risk

We found that a higher number of somatic diseases were associated with an increased hazard of depression over the follow-up, which is in line with the literature (Triolo *et al.*, 2020). However, previous studies employed mostly categorical operationalizations of comorbid diseases (e.g., ≥1 or ≥2 diseases) (Chang *et al.*, 2016; Schoevers *et al.*, 2000) or were carried out in middle-aged individuals (Gerrits *et al.*, 2013; Ronaldson *et al.*, 2021; Yao *et al.*, 2020), making it difficult to compare the results across studies. Overall, this suggests that the general morbidity burden indexed by the crude number of somatic diseases may capture vulnerability to depression in late life. An interaction of multiple overlapping biological (e.g., low-grade chronic inflammation), psychological (e.g., impaired coping strategies) and behavioural (e.g., reduced physical exercise) mechanisms may underpin such association (Gold *et al.*, 2020). This finding corroborates the importance of somatic health in the occurrence of late-life depression and its potential prevention.

### Somatic disease patterns and depression

Our findings showed that specific patterns of coexisting somatic diseases conferred a higher risk of depression. Comparison with previous findings is challenging due to differences in methodologies to derive disease patterns, study population characteristics and depression operationalization (Ronaldson *et al.*, 2021; Yao *et al.*, 2020). Overall, our results align with emerging evidence that delineating disease patterns, particularly with the help of data-driven approaches that detect homogenous groups of individuals exhibiting meaningful differences in somatic burden (Prados-Torres *et al.*, 2014; Skou *et al.*, 2022), can offer valuable insight into future health trajectories (Grande *et al.*, 2021; Ronaldson *et al.*, 2021; Tazzeo *et al.*, 2021; Vetrano *et al.*, 2020). Although there is no consensus on how to define or derive such disease patterns (Skou *et al.*, 2022), a clinical phenotyping of disease combinations could complement measures based on the number of diseases, thus enabling a holistic characterization of older adults and their clinical needs (Whitty *et al.*, 2020).

Compared to individuals without multimorbidity, the *sensory/anaemia, thyroid/musculoskeletal* and *cardiometabolic* patterns were associated with an increased depression incidence, while the *metabolic* and *unspecific* ones were not. Given the specific combinations of over-represented diseases within each pattern, their association with depression is likely to be underpinned by specific mechanisms.

Subjects in the *sensory/anaemia* pattern were primarily characterized by diseases that can lead to visual and hearing impairment, along with anaemia. Although these conditions do not seem to be inherently directly related, common underlying factors, such as chronic kidney disease, nutritional deficiencies or other age-related processes, may explain their co-occurrence (Loscalzo *et al.*, 2022). Epidemiological studies have linked the presence of hearing loss, visual impairment and their co-occurrence to worse depressive trajectories in older people (Armstrong *et al.*, 2022; Brewster *et al.*, 2018; Carrière *et al.*, 2013). Reduced social interactions and social functioning due to sensory impairment have been considered as potential pathways leading to depression development. In addition, depression risk is increased in older people with anaemia (Lee and Kim, 2020), an association that has been attributed to reduced physical function and low cerebral oxygen supply (Gottesman *et al.*, 2012; Penninx *et al.*, 2004).

We found an increased depression risk in individuals presenting with the *thyroid/musculoskeletal* pattern, which was characterized by diseases associated with pain and mobility impairment such as osteoarthritis and dorsopathies. While patients with musculoskeletal disorders present with high rates of comorbid depression, long-term exposure to pain is considered a potential trigger of depression as a psychological reaction, especially in individuals with poorer social and cognitive coping strategies (Gerrits *et al.*, 2014; Goesling *et al.*, 2013; Xue *et al.*, 2020). Further, depression can arise as a side-effect of a long-term use of corticosteroids, which are often prescribed for these conditions. Last, thyroid dysfunctions, especially hypothyroidism, have also been associated with depression in adulthood, although evidence from longitudinal cohorts of older adults is more scarce (Bode *et al.*, 2021, 2022).

Further, the *cardiometabolic* disease pattern, defined by high prevalence of several age-related diseases pertaining mostly to the cardiocirculatory system, was associated with increased depression risk. This finding is in line with previous literature supporting the link between cardiovascular risk factors and diseases with late-life depression, which was formalized in the *vascular depression hypothesis* (Alexopoulos, 2006, 2019). It posits that high cardiovascular and metabolic burden can precipitate late-life depression by entailing cerebrovascular lesions, including white matter hyperintensities, brain infarcts and bleeds (Rensma *et al.*, 2018; van Sloten *et al.*, 2015). Indeed, the increased risk of depression in subjects presenting with this pattern may be explained by the higher cardiovascular burden due to atrial fibrillation, ischemic heart disease and heart failure, along with cerebrovascular disease. Notably, the *metabolic* pattern, which was characterized by cardiovascular risk factors in the absence of either cardio- or cerebrovascular diseases, exhibited no association with depression. This could also suggest differences according to clinical severity, which we attempted to account for by adjusting for malnutrition and by conducting additional analysis in a subpopulation with multimorbidity, revealing a consistent impact of the *cardiometabolic* pattern. Of note, the *cardiometabolic* pattern remained the only one with a significantly increased depression risk (compared to the *unspecific* pattern), which may suggest that in already clinically complex older adults, disease patterns other than the *cardiometabolic* one may discriminate depression risk to a lesser extent. Consequently, our findings underscore a strong link between cardiometabolic diseases and late-life depression.

Last, the *metabolic* and *unspecific* patterns were not associated with increased depression risk. While these may represent milder forms of somatic burden, as suggested above, emerging evidence suggests that disease patterns can evolve into more severe and complex patterns over time (Roso-Llorach *et al.*, 2022; Vetrano *et al.*, 2020), potentially increasing the risk of depression in the future. Therefore, identifying these patterns may represent a critical window of opportunity for preventing the vicious cycle of multimorbidity worsening and depression development.

### Clinical and public health implications

Findings from this study may be of clinical relevance for healthcare providers, as it reinforces the importance of a comprehensive assessment of somatic disease burden in older individuals. Given the complexity of treating depression in older adults with multimorbidity, which is characterized by reduced antidepressant efficacy and increased side-effects, prevention remains a priority (Kok and Reynolds, 2017; Vyas and Okereke, 2020). Characterizing older people by their combinations of co-occurring diseases may, therefore, provide valuable prognostic information and guide preventive and clinical strategies. Individuals with an increased risk may be selectively targeted for interventions to foster physical exercise and social interactions, which may prevent both depression development and multimorbidity progression (Marengoni *et al.*, 2018; Vyas and Okereke, 2020). Further, closer monitoring may be offered to at-risk individuals characterized by specific disease patterns to optimize the clinical control of somatic diseases and promote timely detection of depression. This would further require improved communication and collaboration between primary care and mental health professionals, a critical step towards improved depression care in old age (Langan *et al.*, 2013).

### Strengths and limitations

This study has several strengths including a) a large population-based sample with repeated measures over a 15-year follow-up; b) detailed clinical information used to characterize somatic disease patterns, obtained from multiple health data sources and c) diagnoses of depression derived from an extensive medical and psychiatric assessment. Some limitations require acknowledgement. First, we combined the diagnoses of major and minor depression into a composite outcome. Given the population-based nature of this study, lack of statistical power prevented us from exploring these diagnoses separately. While we acknowledge the clinical differences between these two entities, growing evidence supports the detrimental role of minor depression in old age health, which warrants attention from clinicians and researchers (Meeks *et al.*, 2011). Further, we did not explore the variability of depressive symptoms in relation to disease patterns, and future research may delve into it. Different biological burdens underlying somatic diseases patterns have been associated with specific individual and profiles of depressive symptoms, supporting such hypothesis (Jokela *et al.*, 2016; Penninx, 2017; Penninx *et al.*, 2013; Triolo *et al.*, 2021). Second, dementia development over the follow-up may have led to outcome misclassification by reducing our ability to diagnose depression in cognitively impaired participants, potentially diluting our estimates. Third, our operationalizations of somatic disease burden did not account for disease severity, which was proxied in the longitudinal analyses of depression risk by further adjusting the models for malnutrition, as done in previous studies (Grande *et al.*, 2021; Vetrano *et al.*, 2018b). Fourth, the number and composition of the disease patterns may differ in other settings, thus requiring confirmation. However, these preliminary findings, obtained through extensive heath information, may still provide insight to healthcare professionals faced with the clinical complexity of older people. Fifth, selective dropout due to death or poor health is likely to affect our estimates towards an underestimation. Sixth, unmeasured confounding cannot be ruled out. Last, the external validity of these findings should be critically appraised, given the relatively high socio-economic status and the ethnic homogeneity (white) of SNAC-K participants.

## Conclusion

In this population-based study, somatic disease burden was associated with depression incidence in late life both in terms of co-occurring disease number as well as patterns. Specifically, patterns of *sensory/anaemia, thyroid/musculoskeletal* and *cardiometabolic* multimorbidity conferred increased risk of developing depression in late life, with the effect of the latter remaining even in a subsample of individuals with multimorbidity. These findings further the notion that mental health should be closely monitored among older adults with high somatic disease burden, especially if affected by cardiometabolic multimorbidity.

## Supporting information

Triolo et al. supplementary material Triolo et al. supplementary material

## Data Availability

SNAC-K data (http://www.snac-k.se/) can be accessed by the scientific community upon approval from the SNAC-K management and maintenance committee, and applications can be submitted to Maria Wahlberg (Maria.Wahlberg@ki.se) at the Aging Research Center, Karolinska Institutet. The analytical code is available upon reasonable request.

## Supplementary material

The supplementary material for this article can be found at https://doi.org/10.1017/S2045796024000064.
