# Supplementary material for: Somatic disease burden and depression risk in late life: a community-based study
Source: Epidemiol Psychiatr Sci. 2024 Feb 8;33:e6. doi: 10.1017/S2045796024000064 (PMC10894701; doi:10.1017/S2045796024000064)
Supplement: Triolo et al. supplementary material [file S2045796024000064sup001.docx]

**Figure S1**. Flowchart of the study population.

Dead n= 30

Dropout n= 37

Dead n= 31

Dropout n= 22

SNAC-K Population

N=3363

Analytical sample

n=2904

**Exclusion criteria:**

Depression baseline: n=222

Dementia baseline: n=201

**Missing:**

Depression baseline: n=36

≥78 yrs old N=1217

<78 yrs old N=1687

FU1 (3yrs)

FU2

(6yrs)

FU3 (9yrs)

FU4

(12yrs)

FU5

(15yrs)

Dead n= 133

Dropout n= 211

N=855

Dead n= 235; Dropout n= 127

N=602

Dead n= 181; Dropout n= 72

N=393

Dead n= 172; Dropout n= 37

N=219

Dead n= 140; Dropout n= 34

N=104

Dead n= 101; Dropout n= 14

N=217

N=270

N=261

N=144

Dead n= 30

Dropout n= 44

Not eligible for assessment at FU5 n= 483 *****

Baseline

Dead n= 48

Dropout n= 25

Dead n= 71

Dropout n=117

N=818

N=1343

* Participants aged 72 years old at FU4 are not eligible for assessment at FU5, but will instead be followed up again at FU6. For more information on the study design, we refer to the SNAC-K website (https://www.snac-k.se/).

| **Disease with prevalence>2%** | **Prev., %** | **Disease with prevalence>2%** | **Prev., %** |
| --- | --- | --- | --- |
| Anemia | **12** | Other musculoskeletal and joint diseases | **7** |
| Asthma | **7** | Other neurological diseases | **2** |
| Atrial Fibrillation | **10** | Prostate diseases | **5** |
| Autoimmune disorders | **5** | Solid neoplasms | **11** |
| Blindness and visual impairment | **4** | Thyroid diseases | **11** |
| Bradycardias and conduction diseases | **2** |  |  |
| Cardiac valve diseases | **3** | **Diseases with prevalence<2%** |  |
| Cataract and other lens diseases | **6** | Allergy | **2** |
| Cerebrovascular disease | **8** | Blood and blood forming organ diseases | **1** |
| Chronic kidney diseases | **38** | Chromosomal abnormalities | **0** |
| Colitis and related diseases | **11** | Chronic infectious diseases | **0** |
| COPD, emphysema, chronic bronchitis | **5** | Chronic liver diseases | **0** |
| Deafness, hearing impairment | **11** | Chronic pancreas, biliary tract and gallbladder diseases | **2** |
| Diabetes | **10** | Chronic ulcer of the skin | **1** |
| Dorsopathies | **8** | Ear, nose, throat diseases | **1** |
| Dyslipidemia | **55** | Epilepsy | **1** |
| Esophagus, stomach and duodenum diseases | **5** | Hematological neoplasms | **1** |
| Glaucoma | **6** | Inflammatory bowel diseases | **1** |
| Heart failure | **10** | Multiple sclerosis | **0** |
| Hypertension | **77** | Other digestive diseases | **1** |
| Inflammatory arthropathies | **5** | Other metabolic diseases | **2** |
| Ischemic heart disease | **16** | Other respiratory diseases | **1** |
| Migraine and facial pain syndromes | **2** | Other skin diseases | **0** |
| Obesity | **15** | Parkinson and parkinsonism | **1** |
| Osteoarthritis and other degenerative joint diseases | **15** | Peripheral neuropathy | **2** |
| Osteoporosis | **8** | Peripheral vascular disease | **2** |
| Other cardiovascular diseases | **3** | Venous and lymphatic diseases | **1** |
| Other eye diseases | **6** |  |  |
| Other genitourinary diseases | **3** |  |  |

**Table S1**. Prevalence of 54 somatic diseases in the multimorbid population (2+ diseases; n=2475). Thirty-four diseases with prevalence over 2% were included in the latent class analysis.

| **Number of classes** | **aBIC** |
| --- | --- |
| 3-class model | 47494.65 |
| 4-class model | 47457.40 |
| 5-class model | 47456.42 |
| 6-class model | 47490.73 |

**
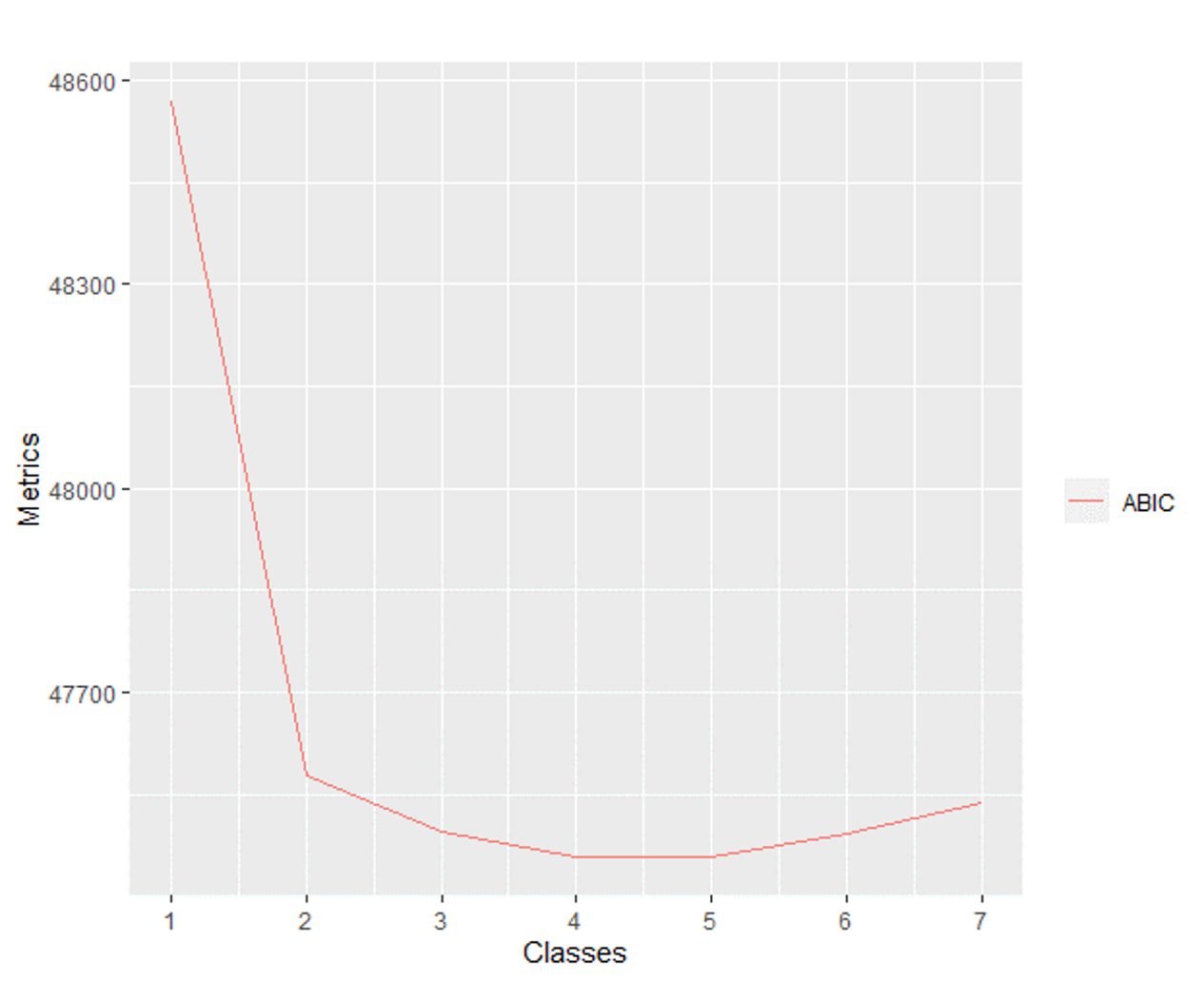
Figure S2**. Scree plot of the adjusted Bayesian Information Criterion (aBIC) of different solutions from latent class analysis.

**Table S2**. Diseases characterizing multimorbidity patterns based on the presence of O/E≥2 and exclusivity>25%.

| **Somatic disease patterns**  (Prevalence in multimorbid subpopulation, N=2475) | **Disease** | **Proportion within the pattern (%)** |
| --- | --- | --- |
| **Unspecific**  (n=716; 29%) | - | - |
| **Metabolic**  (n=369; 15%) | Obesity | 45.3 |
|  | Diabetes | 35.2 |
| **Sensory/Anemia**  (n=514; 21%) | Deafness, hearing impairment | 30.7 |
|  | Anemia | 29.0 |
|  | Cataract and other lens diseases | 17.7 |
|  | Other eye diseases | 17.7 |
|  | Glaucoma | 17.5 |
|  | Blindness, visual impairment | 13.4 |
|  | Other neurological diseases | 4.7 |
| **Thyroid / MSK**  (n=569; 23%) | Osteoarthritis and other degenerative joint diseases | 36.9 |
|  | Thyroid diseases | 26.4 |
|  | Osteoporosis | 20.2 |
|  | Dorsopathies | 19.2 |
|  | Asthma | 16.0 |
| **Cardiometabolic**  (n= 307; 12%) | Heart failure | 75.6 |
|  | Ischemic heart disease | 56.4 |
|  | Atrial fibrillation | 43.3 |
|  | Anemia | 30.6 |
|  | Diabetes | 21.8 |
|  | Cerebrovascular disease | 20.2 |
|  | Other cardiovascular diseases | 18.9 |
|  | Bradycardias and conduction diseases | 13.7 |
|  | Autoimmune diseases | 12.1 |
|  | COPD, emphysema, chronic bronchitis | 12.1 |
|  | Inflammatory arthropathies | 12.1 |
|  | Cardiac valve diseases | 11.7 |
|  | Blindness, visual impairment | 10.1 |

O/E = Observed over expected ratio; MSK: Musculoskeletal; COPD: Chronic Obstructive Pulmonary Disease

**Table S3**. Association between disease patterns and risk of depression after excluding participants with MMSE < 24 or incident cases of dementia in the first 6 years (n=117).

|  | **Model 1**  Events: 193  At risk: 2035 | **Model 2**  Events: 185  At risk: 1973 |
| --- | --- | --- |
| ***Total sample*** |  |  |
| No MM | Reference | Reference |
| Unspecific | **2.00 (1.11-3.62)** | **1.88 (1.04-3.41)** |
| Metabolic | 1.23 (0.59-2.57) | 1.21 (0.58-2.53) |
| Sensory/Anemia | **2.13 (1.10-4.13)** | 1.85 (0.95-3.60) |
| Thyroid/MSK | **2.29 (1.24-4.23)** | **2.06 (1.11-3.82)** |
| Cardiometabolic | **3.76 (1.86-7.62)** | **3.41 (1.65-7.05)** |
|  | **Model 1**  Events: 179  At risk: 1684 | **Model 2**  Events: 171  At risk: 1628 |
| ***Multimorbid subpopulation*** |  |  |
| Unspecific | Reference | Reference |
| Metabolic | 0.62 (0.35-1.10) | 0.66 (0.37-1.17) |
| Sensory/Anemia | 1.07 (0.68-1.69) | 1.99 (0.62-1.57) |
| Thyroid/MSK | 1.14 (0.77-1.69) | 1.10 (0.74-1.64) |
| Cardiometabolic | **1.89 (1.13-3.17)** | **1.84 (1.07-3.17)** |

**Table S4**. Association between disease patterns and risk of depression with additional adjustment for baseline MADRS score (n missing=73).

|  | **Model 1**  Events: 214  At risk: 2108 | **Model 2**  Events: 202  At risk: 2033 |
| --- | --- | --- |
| ***Total sample*** |  |  |
| No MM | Reference | Reference |
| Unspecific | 1.75 (0.98-3.13) | 1.72 (0.96-3.08) |
| Metabolic | 1.08 (0.53-2.18) | 1.04 (0.51-2.13) |
| Sensory/Anemia | **2.07 (1.10-3.89)** | 1.86 (0.98-3.51) |
| Thyroid/MSK | **1.94 (1.07-3.53)** | 1.76 **(**0.96-3.20**)** |
| Cardiometabolic | **3.02 (1.52-6.02)** | **2.69 (1.32-5.48)** |
|  | **Model 1**  Events: 199  At risk: 1765 | **Model 2**  Events: 187  At risk: 1696 |
| ***Multimorbid subpopulation*** |  |  |
| Unspecific | Reference | Reference |
| Metabolic | 0.62 (0.36-1.08) | 0.63 (0.36-1.11) |
| Sensory/Anemia | 1.19 (0.77-1.82) | 1.09 (0.70-1.69) |
| Thyroid/MSK | 1.11 (0.75-1.64) | 1.03 (0.69-1.53) |
| Cardiometabolic | **1.73 (1.04-2.86)** | 1.59 (0.93-2.71) |

**Table S5**. Association between disease patterns and risk of depression with additional adjustment for baseline Antidepressant use (n missing=1).

|  | **Model 1**  Events: 221  At risk: 2151 | **Model 2**  Events: 210  At risk: 2075 |
| --- | --- | --- |
| ***Total sample*** |  |  |
| No MM | Reference | Reference |
| Unspecific | 1.62 (0.92-2.84) | 1.58 (0.90-2.78) |
| Metabolic | 1.07 (0.54-2.14) | 0.99 (0.49-2.00) |
| Sensory/Anemia | **1.91 (1.04-3.53)** | 1.74 (0.94-3.23) |
| Thyroid/MSK | **1.89 (1.67-3.37)** | 1.72 **(**0.96-3.08**)** |
| Cardiometabolic | **2.81 (1.45-5.46)** | **2.58 (1.31-5.10)** |
|  | **Model 1**  Events: 205  At risk: 1797 | **Model 2**  Events: 194  At risk: 1727 |
| ***Multimorbid subpopulation*** |  |  |
| Unspecific | Reference | Reference |
| Metabolic | 0.66 (0.38-1.14) | 0.64 (0.36-1.12) |
| Sensory/Anemia | 1.18 (0.78-1.79) | 1.10 (0.72-1.70) |
| Thyroid/MSK | 1.16 (0.79-1.69) | 1.09 (0.74-1.60) |
| Cardiometabolic | **1.75 (1.07-2.85)** | 1.65 (0.99-2.76) |

STROBE Statement—Checklist of items that should be included in reports of ***cohort studies***

|  | Item No | Recommendation | Page |
| --- | --- | --- | --- |
| **Title and abstract** | 1 | (*a*) Indicate the study’s design with a commonly used term in the title or the abstract | 2 |
|  |  | (*b*) Provide in the abstract an informative and balanced summary of what was done and what was found | 2 |
| Introduction | | |  |
| Background/rationale | 2 | Explain the scientific background and rationale for the investigation being reported | 3-4 |
| Objectives | 3 | State specific objectives, including any prespecified hypotheses | 4 |
| Methods | | |  |
| Study design | 4 | Present key elements of study design early in the paper | 4-5 |
| Setting | 5 | Describe the setting, locations, and relevant dates, including periods of recruitment, exposure, follow-up, and data collection | 4-5 |
| Participants | 6 | (*a*) Give the eligibility criteria, and the sources and methods of selection of participants. Describe methods of follow-up | 4-5 |
|  |  | (*b*) For matched studies, give matching criteria and number of exposed and unexposed | - |
| Variables | 7 | Clearly define all outcomes, exposures, predictors, potential confounders, and effect modifiers. Give diagnostic criteria, if applicable | 5-6 |
| Data sources/ measurement | 8* | For each variable of interest, give sources of data and details of methods of assessment (measurement). Describe comparability of assessment methods if there is more than one group | *5-6* |
| Bias | 9 | Describe any efforts to address potential sources of bias | 4/8 |
| Study size | 10 | Explain how the study size was arrived at | 4-5 |
| Quantitative variables | 11 | Explain how quantitative variables were handled in the analyses. If applicable, describe which groupings were chosen and why | 6-8 |
| Statistical methods | 12 | (*a*) Describe all statistical methods, including those used to control for confounding | 6-8 |
|  |  | (*b*) Describe any methods used to examine subgroups and interactions | 6-8 |
|  |  | (*c*) Explain how missing data were addressed | - |
|  |  | (*d*) If applicable, explain how loss to follow-up was addressed | - |
|  |  | (*e*) Describe any sensitivity analyses | 8 |
| Results | | |  |
| Participants | 13* | (a) Report numbers of individuals at each stage of study—eg numbers potentially eligible, examined for eligibility, confirmed eligible, included in the study, completing follow-up, and analysed | 8 |
|  |  | (b) Give reasons for non-participation at each stage | Sup 1 |
|  |  | (c) Consider use of a flow diagram | Sup 1 |
| Descriptive data | 14* | (a) Give characteristics of study participants (eg demographic, clinical, social) and information on exposures and potential confounders | 8 |
|  |  | (b) Indicate number of participants with missing data for each variable of interest | Table 1 |
|  |  | (c) Summarise follow-up time (eg, average and total amount) | 9 |
| Outcome data | 15* | Report numbers of outcome events or summary measures over time | 9 |
| Main results | 16 | (*a*) Give unadjusted estimates and, if applicable, confounder-adjusted estimates and their precision (eg, 95% confidence interval). Make clear which confounders were adjusted for and why they were included | 9 |
|  |  | (*b*) Report category boundaries when continuous variables were categorized | 9 |
|  |  | (*c*) If relevant, consider translating estimates of relative risk into absolute risk for a meaningful time period | - |
| Other analyses | 17 | Report other analyses done—eg analyses of subgroups and interactions, and sensitivity analyses | 10 |
| Discussion | | |  |
| Key results | 18 | Summarise key results with reference to study objectives | 10 |
| Limitations | 19 | Discuss limitations of the study, taking into account sources of potential bias or imprecision. Discuss both direction and magnitude of any potential bias | 14 |
| Interpretation | 20 | Give a cautious overall interpretation of results considering objectives, limitations, multiplicity of analyses, results from similar studies, and other relevant evidence | 11-13 |
| Generalisability | 21 | Discuss the generalisability (external validity) of the study results | 14 |
| Other information | | |  |
| Funding | 22 | Give the source of funding and the role of the funders for the present study and, if applicable, for the original study on which the present article is based | 15 |

*Give information separately for exposed and unexposed groups.

**Note:** An Explanation and Elaboration article discusses each checklist item and gives methodological background and published examples of transparent reporting. The STROBE checklist is best used in conjunction with this article (freely available on the Web sites of PLoS Medicine at http://www.plosmedicine.org/, Annals of Internal Medicine at http://www.annals.org/, and Epidemiology at http://www.epidem.com/). Information on the STROBE Initiative is available at http://www.strobe-statement.org.
